# *ATP2B4* regulatory genetic variants are associated with mild malaria

**DOI:** 10.1186/s12936-023-04503-8

**Published:** 2023-02-27

**Authors:** Alassane Thiam, Samia Nisar, Mathieu Adjemout, Frederic Gallardo, Oumar Ka, Babacar Mbengue, Gora Diop, Alioune Dieye, Sandrine Marquet, Pascal Rihet

**Affiliations:** 1 Unité d’Immunogénétique, Institut Pasteur de Dakar, Dakar, Senegal; 2 Aix Marseille Univ, INSERM, TAGC, MarMaRa Institute, Marseille, France; 3 Sardar Bahadur Khan Women’s University, Quetta, 1800 Balochistan Pakistan; 4 Service d’Immunologie, Université Cheikh Anta Diop de Dakar, Dakar, Senegal

**Keywords:** Mild malaria, *Plasmodium falciparum*, *ATP2B4*, Regulatory variants

## Abstract

**Background:**

Genome-wide association studies have identified *ATP2B4* as a severe malaria resistance gene. Recently, 8 potential causal regulatory variants have been shown to be associated with severe malaria.

**Methods:**

Genotyping of rs10900585, rs11240734, rs1541252, rs1541253, rs1541254, rs1541255, rs10751450, rs10751451 and rs10751452 was performed in 154 unrelated individuals (79 controls and 75 mild malaria patients). rs10751450, rs10751451 and rs10751452 were genotyped by Taqman assays, whereas the fragment of the *ATP2B4* gene containing the remaining SNPs was sequenced. Logistic regression analysis was used to assess the association between the SNPs and mild malaria.

**Results:**

The results showed that mild malaria was associated with rs10900585, rs11240734, rs1541252, rs1541253, rs1541254, rs1541255, rs10751450, rs10751451 and rs10751452. The homozygous genotypes for the major alleles were associated with an increased risk of mild malaria. Furthermore, the haplotype containing the major alleles and that containing the minor alleles were the most frequent haplotypes. Individuals with the major haplotypes had a significantly higher risk of mild malaria compared to the carriers of the minor allele haplotype.

**Conclusions:**

*ATP2B4* polymorphisms that have been associated with severe malaria are also associated with mild malaria.

**Supplementary Information:**

The online version contains supplementary material available at 10.1186/s12936-023-04503-8.

## Background

Malaria remains one of the most common infectious diseases. According to the World Health Organization (WHO), the estimated number of cases and deaths in 2020 were 241 million and 627,000, respectively [1]. In 2020, 95% and 96% of the cases and deaths occurred in Africa. The most severe forms of the disease are mainly caused by *Plasmodium falciparum*. Host genetic factors have been shown to explain between 20 and 25% of the variations in *P. falciparum* malaria phenotypes [2–4]. *Plasmodium falciparum* can cause asymptomatic infections, mild malaria, or severe malaria, including severe anaemia and cerebral malaria. Genome-wide genetic linkage analyses and genome-wide association studies have been carried out to map genes involved in the control of blood infection levels, mild malaria or severe malaria [5–14].

A large genome-wide association study (GWAS) conducted by Timmann et al. [14] in Ghana identified a new severe malaria locus on chromosome 1q32 within the *ATP2B4* gene. Other GWAS and candidate gene studies conducted in Burkina Faso, Cameroon, Gambia, Kenya, Mali, Malawi, or Tanzania confirmed the association of the *ATP2B4* gene with severe malaria [11, 15, 16]. *ATP2B4* gene encodes for PMCA4, which is the main calcium pump in erythrocytes. Also, it has been hypothesized that genetic variation within *ATP2B4* disrupts the homeostasis of intraerythrocytic calcium concentration and thus, affects the development and structure of intraerythrocytic stages of the parasite [17]. Thus, it is expected that *ATP2B4* genetic variation is associated with parasitaemia and mild malaria attack, which is caused by parasitaemia above a clinical threshold [18]. There was, nevertheless, no report of association analysis of *ATP2B4* single nucleotide polymorphisms (SNP) with parasitaemia or mild malaria.

Most of the genetic association studies reported an association of severe malaria with the tag SNPs rs10900585 or rs4951377, which are in strong linkage disequilibrium with each other. The SNPs rs10751450, rs10751451, and rs10751452 that are in linkage disequilibrium with these tag SNPs were found to influence the expression of *ATP2B4* [17], suggesting that they may be the causal SNPs. More recently, rs10900585, rs10751450, rs10751451, and rs10751452 were reported to be associated with severe malaria in a Senegalese population [19]. Furthermore, 5 new regulatory SNPs (rs11240734, rs1541252, rs1541253, rs1541254, and rs1541255) that disrupt the activity of a new regulatory element were also associated with severe malaria in the Senegalese population [19].

The study aim was to assess the association of the tag SNP rs10900585 and the recently identified regulatory SNPs with mild malaria (MM) in a Senegalese population. Here evidence of an association with mild malaria both at the SNP and haplotype levels is reported.

## Methods

### Study subjects and phenotypes

Mild malaria patient samples (n = 75) were taken at the Principal Hospital of Dakar and the regional hospital of Tambacounda in Senegal on the day of hospital admission [20], whereas control samples (n = 79) were taken from healthy individuals living in Dakar [19]. Additional file 1: Table S1 shows the characteristics of malaria patients and control individuals.

The control individuals were blood donors who did not have any malaria infection (as determined by microscopy) or any other febrile illness, selected at the same period of enrolment. Selected healthy individuals were medically followed by the National Transfusion Center or Hospital Principal more than two years with biological analyses every three months. Only samples from aparasitaemic donors were included in this control group. It should be stressed that the control individuals cannot be considered resistant individuals, since they may be unexposed and uninfected at the time of sampling. The allele and genotype frequencies within the control population rather reflected those within the general population.

All the clinical cases were defined according to WHO criteria [21]. For the patients, the presence of *P. falciparum* infection was determined by an immunoassay enabling the detection of pfHRP2 (Standard diagnostics-Abbott Inc, Chicago, Illinois, USA). According to WHO recommendations, for all subjects, *P. falciparum* parasitaemia was measured by examination of Giemsa-stained blood smears using a magnification × 1000 and by establishing the parasite count per 1000 leukocytes. To obtain reliable results, each slide was examined by two biologists and the mean of the two results was calculated when the difference between them was low. If there was a big difference between the two counts, a third reading was done to reduce the possibility of discordance, and the mean was calculated. The detailed procedure has been described previously [20]. MM patients had fever with *P. falciparum* parasitaemia; the median of parasitaemia was 6516 parasitized red blood cells/μl (25th and 75th percentile 640–37,238 pRBC/μl). MM patients did not show any evidence of impaired consciousness or seizures before and at the time of enrolment. They were surveyed for the two weeks following their enrolment, and there was no case of impaired consciousness or seizures. Blood samples were obtained on the day of hospital admission. The institutional research ethics committee of the University Cheikh Anta Diop approved the study.

### DNA extraction, DNA amplification, and genotyping

Genomic DNA was extracted and further amplified as described [20]. rs10900585, rs11240734, rs1541252, rs1541253, rs1541254 and rs1541255 were genotyped using Sanger sequencing. The fragment of the *ATP2B4* gene containing the polymorphisms was amplified by PCR with 5 μL of whole genomic DNA, 25 μL of Master Mix GoTaq G2 Hot Start (Promega Corporation, Madison, WI USA) according to the manufacturer’s instructions and 3 μL of a mixture of primers sense F: 5′TCAGGCCTAGCTATCAGTTCAG 3′ and antisense R: 5′ CGAGTAGCCGTCCGAAGTC 3′ of 10 μM each. The amplification was carried out in Mastercycler (Eppendorf, Hamburg, Germany) following 35 cycles with a Tm of 60 °C. After amplification, the PCR product was checked on 1.5% agarose gel. The amplicon was then further purified with QIAquick PCR Purification Kit (Qiagen, Hilden, Germany) following the manufacturer’s instructions. Sequencing was performed by GATC (Constance, Germany) from 5 μL of PCR product (20–80 ng / μL) and 5μL of F primer at 5 μM. The chromatograms were read with Chromas 2.6.5 software (South Brisbane, Australia).

rs10751450, rs10751451 and rs10751452 were genotyped by Taqman assays (C_31796478_10, C_31796479_10 and C_31796480_10) respectively. Each reaction well was added with 1.84 μl of H2O, 2.1 μl of 2X Taqman master mix, 0.06 μl of Taqman probes and 1 μl of DNA (10–15 ng/μl). Amplification was performed with polymerase activation at 95 °C for 10 min, followed by 40 cycles of denaturation (95 °C, 15 s) and annealing/extension (60 °C, 1 min). After amplification, allelic identification was performed by the integrated software of the Quantstudio 6 Flex System (Real-time PCR Software v1.3).

### Statistical analysis

SPSS (statistical software version 20) was used to perform data analyses. All the tests were two-tailed. Chi-squared test was used to compare control and patient groups for gender and ethnic groups. Mann–Whitney test was used to compare the age of the controls with that of patients. Logistic regression method that allowed us to take into account potential confounding factors was used to perform genetic association analyses. The Nominal P values, the Odds ratios (OR) and their 95% confidence interval (CI) were calculated. Moreover, the FDR method was used to correct for multiple tests [22]. Genetic association results were considered significant if the FDR was lower than 0.05. Haploview software package was used to test Hardy–Weinberg equilibrium, to construct haplotypes and to calculate their frequency [23].

## Results

### Association between the SNPs and mild malaria

For all the SNPs including the tag SNP (rs10900585) and 8 potential candidate variants (rs11240734, rs1541252, rs1541253, rs1541254, rs1541255, rs10751450, rs10751451 and rs10751452), the observed genotype frequencies in the control group conformed to the Hardy–Weinberg equilibrium (P > 0.5 for all SNPs). Additional file 1: Table S2 shows the minor allele frequency in the studied population and in 1000 genome populations. Additional file 1: Figure S1 shows the linkage disequilibrium r-square coefficients between all the pairs of SNPs. Table 1 shows structural and functional annotations of the SNPs on the basis of public databases [24–27]. Interestingly, there was no functional annotation for rs10900585, whereas the sequences containing the other SNPs bind transcription factors and were annotated as regulatory elements. Table 2 shows relevant Encode and GeneHancer regulatory elements [25, 26], including those containing the studied SNPs. It should be stressed that these potential regulatory elements have been recently experimentally validated [17, 19]. In particular, they were shown to have an enhancer activity, which increases the activity of the main *ATP2B4* promoter [19].

**Table 1:** *ATP2B4* SNP annotation

SNP	Position^**a**^	%genotyped	Structural annotation^b^	Functional annotation^b^		
				Encode	GeneHancer	ReMap^c^
rs10751450 (C > T)	203681817	99.4	Intron	Enhancer	Enhancer	46
rs10751451 (C > T)	203681850	99.4	Intron	Enhancer	Enhancer	49
rs10751452 (T > C)	203681902	99.4	Intron	Enhancer	Enhancer	50
rs11240734 (T > C)	203682696	100	Intron	Enhancer	Enhancer	54
rs1541252 (C > T)	203682799	100	5′ UTR	a. promoter^d^	enhancer	90
rs1541253 (C > T)	203682912	100	5′ UTR	a. promoter	enhancer	149
rs1541254 (G > C)	203683012	100	5′ UTR	a. promoter	enhancer	104
rs1541255 (A > G)	203683013	100	5′ UTR	a. promoter	enhancer	103
rs10900585 (T > G)	203684896	100	Intron	None	None	0

^a^Data represent the position on chromosome 1 according to human hg38 coordinates

^b^SNPs have been annotated using g: Profiler, Encode, GeneHancer and the catalog of ChIP-seq peak ReMap

^c^Number of ChIP-seq peaks using the ReMap catalog. It indicates the ability of the sequence containing the SNP to bind transcription factors

^d^a. promoter means alternative promoter

**Table 2:** Relevant ENCODE and GeneHancer regulatory elements within *ATP2B4* gene

Type	Identifier	Coordinate^a^	Studied SNPs within the regulatory elements
Main promoter	EH38E1413765^b^	203626509–203626856	None
Proximal enhancer	EH38E1413842^b^	203681720–203682066	rs10751450, rs10751451, rs10751452
Alternative promoter	EH38E1413843^b^	203682665–203683013	rs11240734, rs1541252, rs1541253, rs1541254, rs1541255
Enhancer	GH01J203680^c^	203680647–203684029	rs10751450, rs10751451, rs10751452, rs11240734, rs1541252, rs1541253, rs1541254, rs1541255

^a^Data represent the position on chromosome 1 according to human hg38 coordinates

^b^Encode identifier

^c^GeneHancer identifier

The association of the variants with MM was assessed using a logistic regression model. The subjects carrying the homozygous genotype for major alleles were compared with those carrying either the heterozygous genotype or the homozygous genotype for minor alleles, as shown in Table 3. In other words, the association study was performed under a dominant genetic model, which has been proposed in severe malaria studies [11, 14, 15, 19]. A significant association with MM was detected for all the SNPs (P < 0.001) without the effect of any covariate. An association of the SNPs with MM was further detected when considering age as a covariate (P < 0.001); it should be stressed, nevertheless, that controls and mild malaria patients did not differ in age (P = 0.22). Furthermore, the proportion of the Fulani ethnic group was calculated in the control group and in the patient group because membership in the Fulani group reduces the risk of clinical malaria [28]. There was no imbalance between the control group and the patient group with respect to ethnic group membership (P = 0.25). Nevertheless, the ethnic group was included as a covariate in a larger statistical model. The association of the SNPs with mild malaria was confirmed when including age, gender and ethnic groups in the logistic regression model (P < 0.001). Additional file 1: Table S3 shows the detailed results. The homozygous genotypes for the major alleles were found to be associated with an increased risk of developing MM (Table 3). The homozygotes for the major allele had a risk of developing an attack of uncomplicated malaria between 3.17 and 3.62 times higher than carriers of the minor allele. Very similar results were obtained when taking into account age as a covariate (Table 3). Besides, an association study was performed under an additive genetic model. As expected, a significant association of the SNPs with mild malaria was detected (Additional file 1: Table S4). All the association results remained significant after applying an FDR of 5%.

**Table 3:** Association of *ATP2B4* SNPs with mild malaria in Senegalese population

	Position^a^	Minor allele	Risk genotype	Odd ratio	95% CI	P value
rs10751450 (C > T)	203681817	T	CC	3.55^b^ (3.52)^c^	1.83–6.91^b^ (1.78–6.95)^c^	0.0002^b^ (0.0003)^c^
rs10751451 (C > T)	203681850	T	CC	3.35 (3.32)	1.72–6.51 (1.69–6.53)	0.0003 (0.0005)
rs10751452 (T > C)	203681902	C	TT	3.17 (3.13)	1.64–6.14 (1.60–6.15)	0.0006 (0.0009)
rs11240734 (T > C)	203682696	C	TT	3.42 (3.39)	1.76–6.64 (1.72–6.66)	0.0003 (0.0004)
rs1541252 (C > T)	203682799	T	CC	3.42 (3.39)	1.76–6.64 (1.72–6.66)	0.0003 (0.0004)
rs1541253 (C > T)	203682912	T	CC	3.42 (3.39)	1.76–6.64 (1.72–6.66)	0.0003 (0.0004)
rs1541254 (G > C)	203683012	C	GG	3.62 (3.60)	1.86–7.05 (1.83–7.10)	0.0001 (0.0002)
rs1541255 (A > G)	203683013	G	AA	3.62 (3. 60)	1.86–7.05 (1.83–7.10)	0.0001 (0.0002)
rs10900585(T > G)	203684896	G	TT	3.42^b^ (3.39)^c^	1.76–6.6^b^ (1.72–6.66)^c^	0.0003^b^ (0.0004)^c^

^a^Data represent the position on chromosome 1 according to human hg38 coordinates

^b^Results of the logistic regression analysis without any covariate

^c^Results of the logistic regression analysis when taking into account age as a covariate

### Association between haplotypes and mild malaria

This analysis focused on the potential functional variants. First, a haplotype analysis was conducted under the dominant model, which combined rs11240734, rs1541252, rs1541253, rs1541254 and rs1541255. In all, 3 haplotypes were observed with various frequencies ranging from 0.025 to 0.570 in the control group (Table 4). The 2 most frequent haplotypes were that containing the major alleles (TCCGA) and that containing the minor alleles (CTTCG) in both control and mild malaria groups. The frequency of the former haplotype was 0.570 and 0.784 in the control group and the mild malaria group, respectively. In contrast, the frequency of the later haplotype was 0.405 and 0.209, respectively. The frequency of the third haplotype was lower than 0.025 in both groups. Also, the two most frequent haplotypes were further studied. The homozygotes for the haplotype containing the major alleles (TCCGA) were compared with the carriers of the haplotype containing the minor alleles (CTTCG). The analysis showed that individuals with the major allele haplotype had a 3.4 higher risk of mild malaria compared to the carrier of the minor allele haplotype (Table  5). Very similar results were obtained after including age or age, gender and ethnic groups as covariates in the logistic regression model (Additional file 1: Tables S3 and S4).

**Table 4:** Haplotype frequency in the Senegalese population

Study subjects	5 SNP haplotype^a^	Frequency	8 SNP haplotype^b^	Frequency
Control	TCCGA CTTCG TCCCA	0.570 0.405 0.025	CCTTCCGA TTCCTTCG CCTTCCCA TCTTCCGA CCTCTTCG CTTTCCGA	0.544 0.392 0.025 0.019 0.013 0.006
Mild malaria	TCCGA CTTCG TCCCA	0.784 0.209 0.007	CCTTCCGA TTCCTTCG TTCCTTGA CCTCTTCG	0.784 0.203 0.007 0.007

^a^rs11240734, rs1541252, rs1541253, rs1541254 and rs1541255 were included in the haplotype analysis

^b^rs10751450, rs10751451 rs10751452, rs11240734, rs1541252, rs1541253, rs1541254 and rs1541255 were included in the haplotype analysis

**Table 5:** Haplotype association analysis in the Senegalese population

Major allele haplotype	Minor allele haplotype	Risk genotype	OR	95% CI	P value
TCCGA	CTTCG	TCCGA/TCCGA^a^	3.40^c^ (3.45)^d^	1.73–6.69^c^ (1.73–6.86)^d^	0.0003^c^ (0.0004)^d^
CCTTCCGA	TTCCTTCG	CCTTCCGA/CCTTCCGA^b^	3.53 (3.51)	1.78–7.02 (1.74–7.08)	0.0003 (0.0004)

^a^rs11240734, rs1541252, rs1541253, rs1541254 and rs1541255 were included in the analysis

^b^rs10751450, rs10751451 rs10751452, rs11240734, rs1541252, rs1541253, rs1541254 and rs15412552 were included in the analysis. OR, odds ratio; CI, confidence interval

^c^Results of the logistic regression analysis without any covariate

^d^Results of the logistic regression analysis when taking into account age as a covariate

The haplotype analysis was further carried out under the dominant model by including rs10751450, rs10751451, rs10751452, rs11240734, rs1541252, rs1541253, rs1541254 and rs1541255. Seven haplotypes were observed (Table  4). Again, the most frequent haplotypes were that containing the major alleles (CCTTCCGA) and that containing the minor alleles (TTCCTTCG). The frequency of the major allele haplotype was 0.544 and 0.784 in the control group and the mild malaria group, whereas that of the minor allele haplotype was 0.392 and 0.203 in the control group and the mild malaria group, respectively. The frequency of the other haplotypes was lower than 0.025 in both groups. Also, these haplotypes were not considered in further analysis. Homozygotes for the major haplotypes were found 3.5 times more likely to have mild malaria than carriers of the minor allele haplotype when including covariates in the statistical model or not (Additional file 1: Table S3). Noticeably, a significant association was also found between haplotypes and mild malaria using an additive genetic model (Additional file 1: Table S4). All the association results remained significant after applying an FDR of 5%.

## Discussion

GWAS have identified *ATP2B4* as a severe malaria locus [11, 14]. In particular, rs10900585 has been associated with severe malaria in several independent populations, and a meta-analysis further confirmed this association [19]. Eight regulatory SNPs in linkage disequilibrium with rs10900585 were further identified, and evidence of the association between these regulatory SNPs and severe malaria in Senegal was reported [19]. Here the aim of the study was to assess the association of these SNPs with mild malaria in Senegal.

The results showed the association of mild malaria with rs10751450, rs10751451, rs10751452, rs11240734, rs1541252, rs1541253, rs1541254 and rs1541255. Homozygotes for the major alleles had almost a threefold increased risk of mild malaria, compared to carriers of the minor alleles. Similarly, individuals with the major allele haplotype had an increased risk of mild malaria. Interestingly, the major alleles of those SNPs have been associated with severe malaria [19], whereas carriers of the minor alleles were associated with protection against severe malaria. Besides, individuals with the minor allele haplotype were shown to have a reduced expression of PMCA4b [19, 29].

It has been proposed that the reduction of the expression of *ATP2B4* that encodes for PMCA4 causes an increased intracellular calcium concentration, increased MCHC, erythrocyte dehydration and finally a reduced parasite level [17, 19]. Very recently, Joof et al. [29] investigated the effect of *ATP2B4* polymorphisms on the in vitro growth of parasites. They found a reduced parasite growth in red blood cells taken from homozygotes carriers of the minor haplotype, whereas they did not find any effect of those polymorphisms on neither invasion nor adhesion. In addition, Pance et al. [30] showed that the inhibition of the expression of PMCA4 had a slight effect on the in vitro growth of parasites in human stem cell-derived erythroid cells. In contrast, *ATP2B4* gene targeting in mice infected by *Plasmodium berghei* strain ANKA did not alter parasitaemia, although it protected against cerebral malaria [31]. The authors found an increased expression of PMCA1 in *ATP2B4*−/− mice and, therefore, suggested that PMCA1 and other calcium channels may compensate for a low expression of *ATP2B4* and a low level of PMCA4 at the cell surface. These results indicate the need to assess the effect of *ATP2B4* variants on parasitaemia measured in vivo in subjects living in malaria-endemic areas.

## Conclusions

*ATP2B4* minor allele haplotype that protects against severe malaria also protects against mild malaria in a Senegalese population. This is consistent with the biological model based on an impaired expression of PMCA4 at the cell surface leading to an accumulation of intracellular calcium that activates the GARDOS channel causing erythrocyte dehydration and a reduction of the parasite growth. However, further studies are needed to evaluate the association between *ATP2B4* polymorphisms and parasitaemia.

## Supplementary Information

**Additional file 1: Figure S1.** Linkage disequilibrium (LD) (r-squared) map for the *ATP2B4* variants in the studied population. r-squared values ranged from 0.83 to 1. r-squared values were equal to 1 for the haplotype block containing rs11240734, rs1541252, rs1541253. **Table S1**. Characteristics of study subjects. **Table S2.** SNP minor allele frequency in the study population and in other African populations. The position is the position on chromosome 1 according to human hg38 coordinates. **Table S3.** Genetic association results under the genetic dominant model. Haplotype contains the studied SNPs but rs10900585, which is not functional. **Table S4.** Genetic association results under the genetic additive model. Haplotype contains the studied SNPs but rs10900585.

## Data Availability

The full data used may be released upon reasonable request to the corresponding authors.

## Abbreviations

GWAS: Genome-wide association study
MM: Mild malaria
OR: Odd ratio
SNP: Single nucleotide polymorphism
