# Supplementary material for: ATP2B4 regulatory genetic variants are associated with mild malaria
Source: Malar J. 2023 Feb 27;22:68. doi: 10.1186/s12936-023-04503-8 (PMC9972758; doi:10.1186/s12936-023-04503-8)
Supplement: Supplementary file 1 — Additional file 1: Figure S1. Linkage disequilibrium (LD) (r-squared) map for the ATP2B4 variants in the studied population. r-squared values ranged from 0.83 to 1. r-squared values were equal to 1 for the haplotype block containing rs11240734, rs1541252, rs1541253. Table S1. Characteristics of study subjects. Table S2. SNP minor allele frequency in the study population and in other African populations. The position is the position on chromosome 1 according to human hg38 coordinates. Table S3. Genetic association results under the genetic dominant model. Haplotype contains the studied SNPs but rs10900585, which is not functional. Table S4. Genetic association results under the genetic additive model. Haplotype contains the studied SNPs but rs10900585. [file 12936_2023_4503_MOESM1_ESM.pdf]

Figure S1

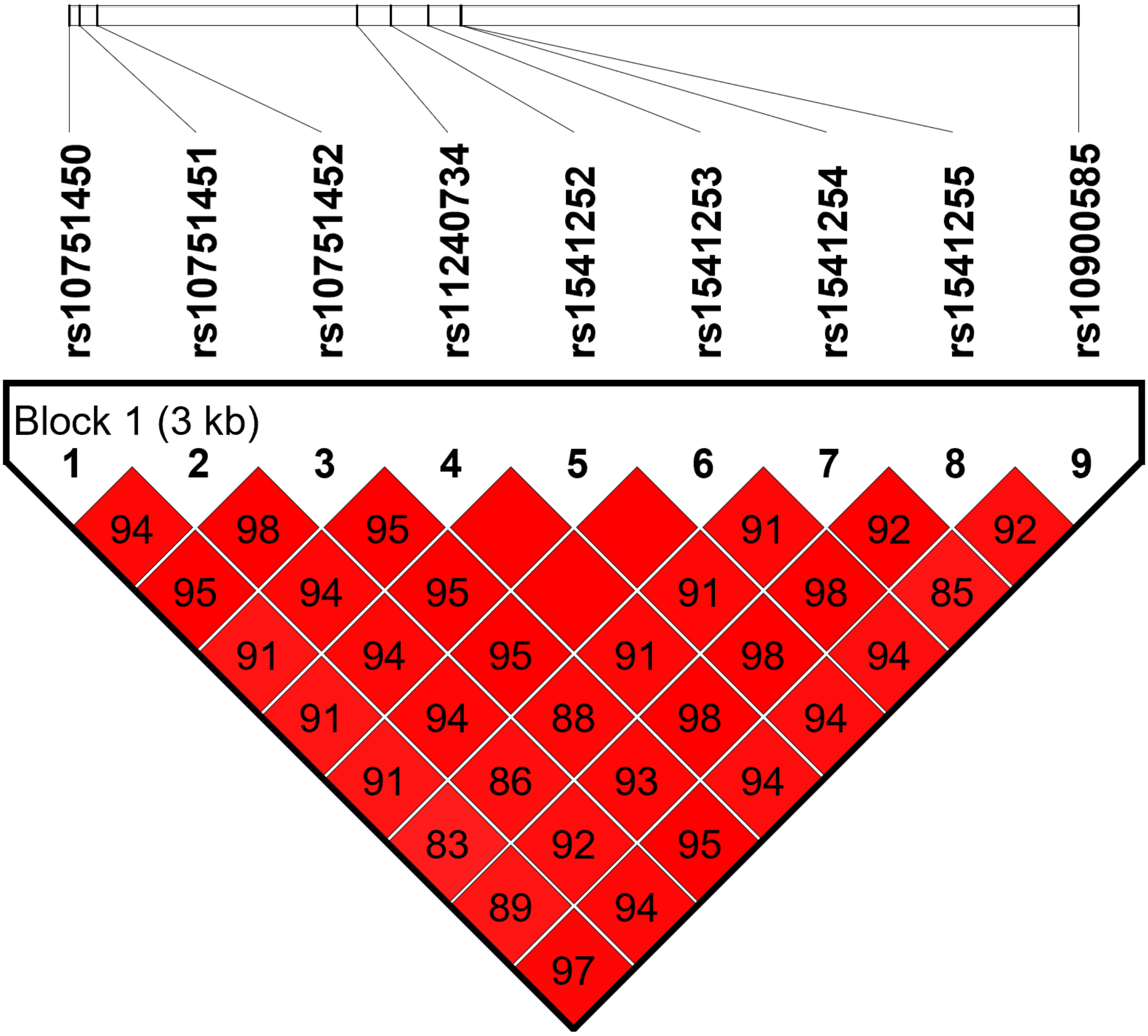
rs10751450
rs10751451
rs10751452
rs11240734
rs1541252
rs1541253
rs1541254
rs1541255
rs10900585
Block 1 (3 kb)
1
2
3
4
5
6
7
8
9
94
98
95
91
92
92
95
94
95
91
98
85
91
94
95
91
98
94
91
94
88
98
94
91
86
93
94
83
92
95
89
94
97

Table S1: Characteristics of study subjects

	Control group (N = 79)	Mild malaria (N = 75)
**Gender**		
N	78	73
Female	38	34
Male	40	39
**Ethnic group**[a]		
N	75	68
Bambara	8	5
Peulh/Fulani	25	29
Serrere	13	7
Wolof	22	16
Others	7	11
**Age**[b]		
N	76	66
Median	33.0	28.5
(25th and 75th percentile)	(23.0-50.3)	(16.5-42.3)
**Haematological data**[b]		
- Red blood cells ($x10^6/\mu L$)		
N	50	66
Median	4.5	4.0
(25th and 75th percentile)	(4.0-5.0)	(3.5-4.6)
- Haematocrit (%)		
N	50	66
Median	36.3	34.6
(25th and 75th percentile)	(33.2-41.2)	(29.8 – 38.4)
- Haemoglobin (g/dL)		
N	50	66
Median	12.1	11.1
(25th and 75th percentile)	(11.2-13.9)	(9.8–12.4)
- Leukocytes ($x10^3/\mu L$)		
N	50	66
Median	6.0	7.9
(25th and 75th percentile)	(4.8-7.2)	(5.1–10.8)
- Platelets ($x10^3/\mu L$)		
N	50	66
Median	260	183
(25th and 75th percentile)	(222–338)	(109-237)
**Successfully genotyped individuals (N)**		
For rs10900585, rs10900585, rs11240734, rs1541252, rs1541253, rs1541254, rs1541255	79	75
For rs10751450, rs10751451 and rs10751452	79	74

[a]Ethnic groups are shown. Other ethnic groups that are Diola, Cap-vert, Soninke, and Soce groups were rare (n≤7). The ethnic group was unknown for four control subjects and seven malaria patients.
[b]Median and (25th and 75th percentile) are shown

Table S2: SNP minor allele frequency in the study population and in other African populations

SNP	Position	number of genotyped individuals/total samples	MAF in the Senegalese population			MAF in 1000 Genome populations	
			Control group	Mild malaria group	Whole population	Gambia	Africa
rs10751450 (C>T)	203681817	153/154	0.411	0.209	0.314	0.385	0.426
rs10751451 (C>T)	203681850	153/154	0.399	0.209	0.307	0.310	0.366
rs10751452 (T>C)	203681902	153/154	0.392	0.209	0.304	0.310	0.365
rs11240734 (T>C)	203682696	154/154	0.405	0.220	0.315	0.310	0.365
rs1541252 (C>T)	203682799	154/154	0.405	0.220	0.315	0.310	0.365
rs1541253 (C>T)	203682912	154/154	0.405	0.220	0.315	0.310	0.365
rs1541254 (G>C)	203683012	154/154	0.430	0.220	0.328	0.310	0.365
rs1541255 (A>G)	203683013	154/154	0.405	0.213	0.312	0.310	0.365
rs10900585 (T>G)	203684896	154/154	0.411	0.213	0.315	0.363	0.418

Table S3: Genetic association results under the genetic dominant model

		Without covariate			Age as a covariate			Age, gender, ethnic group as covariates		
	**Risk Genotype**	**Odd ratio**	**95% CI**	**P value**	**Odd ratio**	**95% CI**	**P value**	**Odd ratio**	**95% CI**	**P value**
rs10751450 (C>T)	CC	3.55	1.83-6.91	0.0002	3.52	1.78-6.95	0.0003	3.45	1.73-6.86	0.0004
rs10751451 (C>T)	CC	3.35	1.72-6.51	0.0003	3.32	1.69-6.53	0.0005	3.25	1.64-6.44	0.0007
rs10751452 (T>C)	TT	3.17	1.64-6.14	0.0006	3.13	1.60-6.15	0.0009	3.06	1.55-6.06	0.001
rs11240734 (T>C)	TT	3.42	1.76-6.64	0.0003	3.39	1.72-6.66	0.0004	3.32	1.68-6.57	0.0006
rs1541252 (C>T)	CC	3.42	1.76-6.64	0.0003	3.39	1.72-6.66	0.0004	3.32	1.68-6.57	0.0006
rs1541253 (C>T)	CC	3.42	1.76-6.64	0.0003	3.39	1.72-6.66	0.0004	3.32	1.68-6.57	0.0006
rs1541254 (G>C)	GG	3.62	1.86-7.05	0.0001	3.60	1.83-7.10	0.0002	3.53	1.78-7.01	0.0003
rs1541255 (A>G)	AA	3.62	1.86-7.05	0.0001	3.60	1.83-7.10	0.0002	3.53	1.78-7.01	0.0003
rs10900585 (T>G)	TT	3.42	1.76-6.60	0.0003	3.39	1.72-6.66	0.0004	3.32	1.68-6.57	0.0006
Haplotype[a]	CCTTCCGA/CCTTCCGA	3.53	1.78-7.02	0.0003	3.51	1.74-7.08	0.0004	3.59	1.77-7.27	0.0004

[a]rs10751450, rs10751451 rs10751452, rs11240734, rs1541252, rs1541253, rs1541254 and rs1541255 were included in haplotype analysis

Table S4: Genetic association results under the genetic additive model

		Without covariate			Age as a covariate			Age, gender, ethnic group as covariates		
	**Risk allele**	**Odd ratio**	**95% CI**	**P value**	**Odd ratio**	**95% CI**	**P value**	**Odd ratio**	**95% CI**	**P value**
rs10751450 (C>T)	C	2.69	1.57-4.59	0.0003	2.65	1.54-4.57	0.0004	2.60	1.51-4.48	0.0006
rs10751451 (C>T)	C	2.56	1.50-4.39	0.0006	2.53	1.47-4.35	0.0008	2.48	1.44-4.27	0.0011
rs10751452 (T>C)	T	2.47	1.45-4.20	0.0009	2.43	1.41-4.15	0.0010	2.38	1.39-4.08	0.0016
rs11240734 (T>C)	T	2.45	1.45-4.15	0.0008	2.42	1.42-4.10	0.0010	2.37	1.39-4.03	0.0015
rs1541252 (C>T)	C	2.45	1.45-4.15	0.0008	2.42	1.42-4.10	0.0010	2.37	1.39-4.03	0.0015
rs1541253 (C>T)	C	2.45	1.45-4.15	0.0008	2.42	1.42-4.10	0.0010	2.37	1.39-4.03	0.0015
rs1541254 (G>C)	G	2.48	1.51-4.08	0.0004	2.42	1.45-4.03	0.0007	2.33	1.39-3.91	0.0014
rs1541255 (A>G)	A	2.54	1.50-4.29	0.0005	2.42	1.46-4.03	0.0008	2.45	1.44-4.18	0.0010
rs10900585 (T>G)	T	2.64	1.55-4.48	0.0004	2.60	1.52-4.46	0.0005	2.55	1.49-4.39	0.0007
Haplotype[a]	CCTTCCGA	2.64	1.53-4.57	0.0005	2.6	1.50-4.52	0.0007	2.62	1.50-4.57	0.0007

[a]rs10751450, rs10751451 rs10751452, rs11240734, rs1541252, rs1541253, rs1541254 and rs1541255 were included in haplotype analysis
